# Partitioning clustering algorithms for protein sequence data sets

**DOI:** 10.1186/1756-0381-2-3

**Published:** 2009-04-02

**Authors:** Sondes Fayech, Nadia Essoussi, Mohamed Limam

**Affiliations:** 1 Department of Computer Science, LARODEC Laboratory, Higher Institute of Management, University of Tunis, Tunis, Tunisia

## Abstract

**Background:**

Genome-sequencing projects are currently producing an enormous amount of new sequences and cause the rapid increasing of protein sequence databases. The unsupervised classification of these data into functional groups or families, clustering, has become one of the principal research objectives in structural and functional genomics. Computer programs to automatically and accurately classify sequences into families become a necessity. A significant number of methods have addressed the clustering of protein sequences and most of them can be categorized in three major groups: hierarchical, graph-based and partitioning methods. Among the various sequence clustering methods in literature, hierarchical and graph-based approaches have been widely used. Although partitioning clustering techniques are extremely used in other fields, few applications have been found in the field of protein sequence clustering. It is not fully demonstrated if partitioning methods can be applied to protein sequence data and if these methods can be efficient compared to the published clustering methods.

**Methods:**

We developed four partitioning clustering approaches using Smith-Waterman local-alignment algorithm to determine pair-wise similarities of sequences. Four different sets of protein sequences were used as evaluation data sets for the proposed methods.

**Results:**

We show that these methods outperform several other published clustering methods in terms of correctly predicting a classifier and especially in terms of the correctness of the provided prediction. The software is available to academic users from the authors upon request.

## Background

In bioinformatics, the number of protein sequences is more than half a million, and it is necessary to find meaningful partitions of them in order to detect their functions. Early approaches of comparing and grouping protein sequences are alignment methods. In fact, pair-wise alignment is used to compare and to cluster sequences. There are two types of pair-wise sequence alignments, local and global [1,2]. Smith and Waterman local alignment algorithm [3] helps in finding conserved amino acid patterns in protein sequences. Needleman and Wunsch global alignment algorithm [4] attempts are made to align the entire sequence using as many characters as possible, up to both ends of each sequence. In order to cluster a large data set of proteins into meaningful clusters, the pair-wise alignment is computationally expensive because of the large number of comparisons carried out. In fact, each protein of the data set should be compared to all others of the data set.

For this reason the pair-wise alignment methods are not efficient to cluster a large set of data. These approaches do not consider the fact that the data set can be too large and may not fit into the main memory of some computers.

The main objective of the unsupervised learning technique is to find a natural grouping or meaningful partition using a distance function [5,6]. Clustering is a technique which has been extensively applied in a variety of fields related to life science and biology. In sequence analysis, clustering is used to group homologous sequences into gene or protein families.

Many methods are currently available for the clustering of protein sequences into families and most of them can be categorized in three major groups: hierarchical, graph-based and partitioning methods. Among these various methods, most are based on hierarchical or graph-based techniques and they were successfully established. In fact, COG [7] uses a hierarchical merging of clusters and a manual validation to prevent chaining of multi-domain families. ProtoNet [8] uses a special metric as described by Sasson et al. [9] to merge clusters. Picasso [10] uses multiple alignments as profiles which are then merged hierarchically. ClusTr [11] and GeneRage [12] use standard single linkage clustering approaches. SYSTERS [13] combines hierarchical clustering with graph-based clustering. ProtoMap [14] and N-cut [15,16] methods use graph-based clustering approaches. ProClust [17] uses an extension of the graph-based clustering approach proposed by [18]. ProClust algorithm is based on transitivity criterion and it is capable of handling multi-domain proteins. TribeMCL [19] applies the Markov clustering approach (MCL) described by Van Dongen [20]. This method operates on a graph that contains similarity information obtained by pair-wise alignment of sequences.

A small amount of partitioning techniques is used in the protein sequence clustering field. Guralnik and Karypis [21] have proposed one method based on a standard k-means approach where proteins are represented by vectors. However, no tool or database resulting from this interesting work has been made available to the scientific community. JACOP [22] uses the partitioning algorithm implemented under the name PAM (Partitioning Around Medoids) in the R statistical package [23]. JACOP is based on a random sampling of sequences into groups. It is available on the MyHits platform [24] where user can submit his own data set. Methods presented bellow are not generally tools since they cannot be applied to cluster a user-provided data set. In fact, several of these methods have been applied to large known data sets and user can only consult the resulting classifications stored in databases. Among the protein sequence clustering methods defined bellow only ProClust, TribeMCL and JACOP are accessed by the community and user can classify his own sequence set.

The main idea here is to design and develop efficient clustering algorithms based on partitioning techniques, which are not very investigated in protein sequence clustering field, in order to cluster large sets of protein sequences. In fact, the number of protein sequences available now is very important (in the order of millions) and hierarchical methods are computationally very expensive so they cannot be extended to cluster large protein sets. However, partitioning methods are very simple and more appropriate to cluster large data sets [22]. For these reasons, we propose here new clustering algorithms based on partitioning techniques which aim to find meaningful partitions, to improve the classification's quality and to reduce the computation time compared to the published clustering tools, ProClust, TribeMCL and JACOP, on different data sets.

Several partitioning clustering algorithms have been proposed in literature. K-means [23,25-27] is a standard partitioning clustering method based on K centroids of a random initial partition which is iteratively improved. LEADER [28,29] is an incremental partitioning clustering algorithm in which each of the K clusters is represented by a leader. CLARA (Clustering LARge Applications) [23] is a partitioning algorithm based on a combination of a sampling approach and the PAM algorithm. CLARANS (Clustering Large Applications based on RANdomized Search) [30] algorithm views the process of finding optimal medoids as searching through a certain graph, in which each node represents a set of medoids.

We adapted the partitioning algorithms cited bellows to protein sequence data sets. These proposed algorithms are named: Pro-Kmeans, Pro-LEADER, Pro-CLARA and Pro-CLARANS. Performance measures are used to evaluate the proposed methods and to compare them with ProClust, TribeMCL and JACOP results.

## Methods

### Algorithms Implementation

To facilitate subsequent discussion, the main symbols used through the paper and their definitions are summarized in Table 1.

**Table 1:** Summary of symbols and definitions

**Symbols**	**Definitions**
D	Data set of protein sequences to be clustered
K	Number of clusters
n	Number of proteins in *D*
O_i_	a protein sequence *i *in *D*
q	Number of iterations

The main objective in the proposed algorithms Pro-Kmeans, Pro-LEADER, Pro-CLARA and Pro-CLARANS is to produce K clusters from a data set D of n protein sequences, so that the objective function f(V) is maximized.

f(V) is the global score function that evaluates the clustering quality and it is as follows

(1)

Where R_i _is the centroid of the group i for which belong the object O_j _and Score (O_j_, R_i_) is the alignment score of the protein sequences O_j _and R_i_, calculated as follows

(2)

Where *S (A*_*i*_, *B*_*j*_*) *is the substitution score of the amino acid *A*_*i *_by *B*_*j *_as determined from a scoring matrix and *g(n) *is the total cost of penalties for a gap length *n*. The gap is defined as follows

(3)

Where P_o _is the gap opening penalty and P_e _is the gap extension penalty.

We chose Smith and Waterman local alignment algorithm for computing alignment score. The choice of this algorithm was motivated by the sensitivity for low-scoring alignments [31] compared to heuristic algorithms such as FASTA [32] and BLAST [33], and by execution time [34] compared to Needleman and Wunsch global alignment algorithm.

We present here Pro-Kmeans, Pro-LEADER, Pro-CLARA and Pro-CLARANS partitioning clustering algorithms for protein sequence sets.

#### Pro-Kmeans algorithm

The Pro-Kmeans algorithm proposed here, starts by a random partition of the data set D into K clusters and then uses the Smith Waterman algorithm to compare proteins of each cluster S_i__(i ∈ [1..K]) _and to compute SumScore(S_i_, O_j_) of each protein j in S_i _as follows

(4)

Where *m *is the size of the subset *S*_*i*_, for which belongs the object *O*_*j*_.

**Figure 1:**
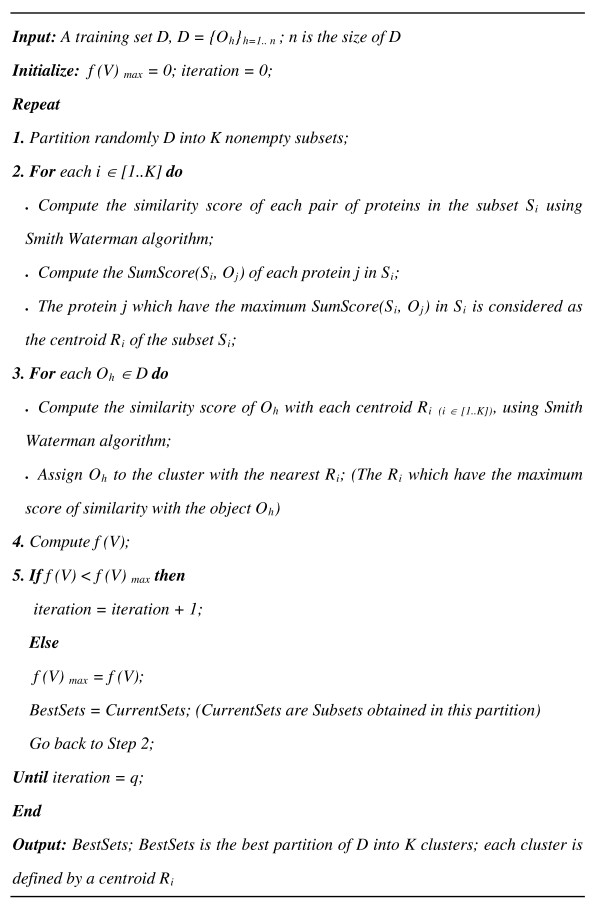
**Pseudo code for Pro-Kmeans algorithm**.

The sequence *O*_*j *_in each cluster *S*_*i *_which has the maximum *SumScore*(*S*_*i*_, *O*_*j*_) is considered as the centroid *R*_*i *_of the cluster. The Smith Waterman algorithm is used here also to compare each protein *O*_*h *_of the data set *D *with centroids and to assign the object to the nearest cluster where the *R*_*i *_have the maximum score of similarity with the object *O*_*h*_. Pro-Kmeans proceeds to this procedure for a number of times, *q*, in order to maximize the *f(V) *function. Input parameters are the number of clusters, *K*, and of iterations, *q*, and as outputs the algorithm returns the best partition of the training base *D *and the center, or mean, of each cluster *S*_*i*_. Pro-Kmeans algorithm is illustrated in Figure 1.

#### Pro-LEADER algorithm

**Figure 2:**
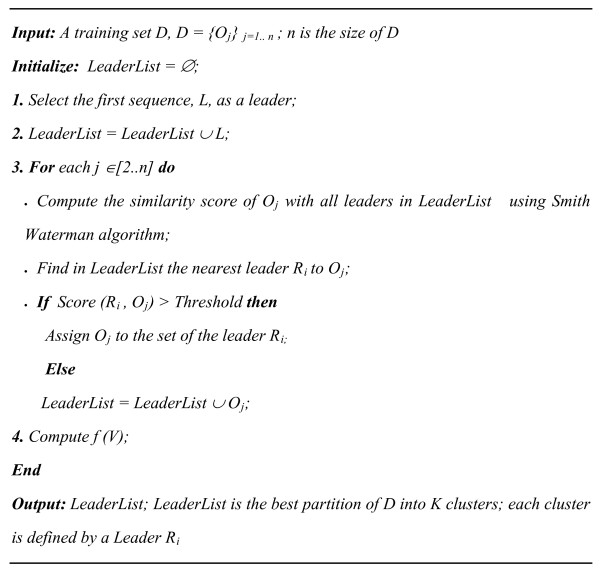
**Pseudo code for Pro-LEADER algorithm**.

Pro-LEADER is an incremental algorithm which selects the first sequence of the data set *D *as the first leader, and use the Smith Waterman algorithm to compute the similarity score of each sequence in *D *with all leaders. The algorithm detects the nearest leader *R*_*i *_to each sequence *O*_*j *_and compares the score, *Score(R*_*i*_, *O*_*j*_*)*, with a pre-fixed *Threshold*. If the similarity score of *R*_*i *_and *O*_*j*_, is more than the *Threshold*, *O*_*j *_is considered as a new leader and if not, the sequence *O*_*j *_is assigned to the cluster defined by the leader *R*_*i*_. Pro-LEADER is thus an incremental algorithm in which each of the *K *clusters is represented by a leader. The *K *clusters are generated using a suitable *Threshold *value. Pro-LEADER aims also to maximize the *f(V) *function. Input parameter is the similarity score *Threshold *to consider an object *O*_*j *_as a new leader, and as outputs the algorithm returns the best partition of the training base *D *and the *K *leaders of the obtained clusters. The Pro-LEADER algorithm is fast, requiring only one pass through the data set *D*. Pro-LEADER algorithm is depicted in Figure 2.

#### Pro-CLARA algorithm

Pro-CLARA relies on the sampling approach to handle large data sets [23]. Instead of finding medoids for the entire data set, Pro-CLARA algorithm draws a small sample *S *of 40 + 2*K *sequences from the data set *D*. To generate an optimal set of medoids for this sample, Pro-CLARA applies the proposed PAM algorithm for protein sequence data sets, Pro-PAM algorithm,

The Pro-PAM algorithm proposed here, selects randomly K sequences from the data set as clusters, and then use the Smith Waterman algorithm to compute the total score TS_ih _of each pair of selected sequence R_i _and non selected sequence O_h_. TS_ih _is as follows

(5)

Where *S*_*jih *_is the differential score of each pair of non-selected object *O*_*h *_in *D *and selected object *R*_*i*(*i *∈ [1..*K*]) _with all non-selected objects *O*_*j *_in *D. S*_*jih *_is as follows

(6)

**Figure 3:**
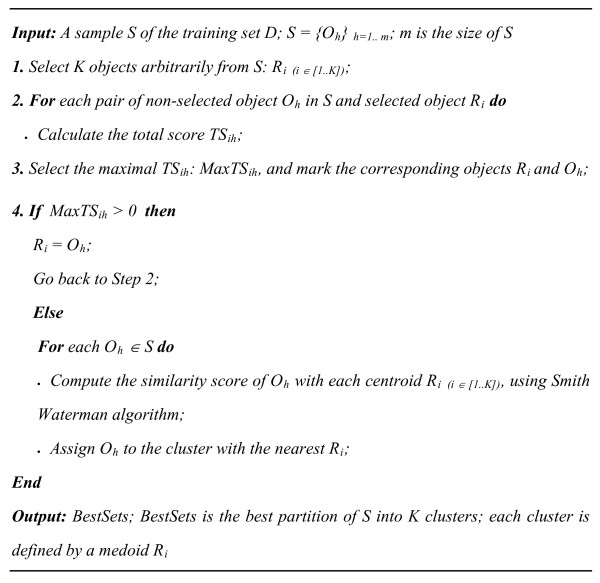
**Pseudo code for Pro-PAM algorithm**.

Pro-PAM selects the maximal *TS*_*ih*_, *MaxTS*_*ih*_. If *MaxTS*_*ih *_is positive, the corresponding non selected sequence *O*_*h *_will be selected, otherwise Smith Waterman algorithm is used to compare each protein *O*_*h *_of the data set with all medoids *R*_*i*(*i *∈ [1..*K*])_, and to assign the sequence *O*_*h *_to the nearest cluster. Input parameter of Pro-PAM is the number of clusters, *K*, and as outputs the algorithm returns the best partition of the protein sequence base and the medoid of each cluster. Pro-PAM algorithm is depicted in Figure 3.

**Figure 4:**
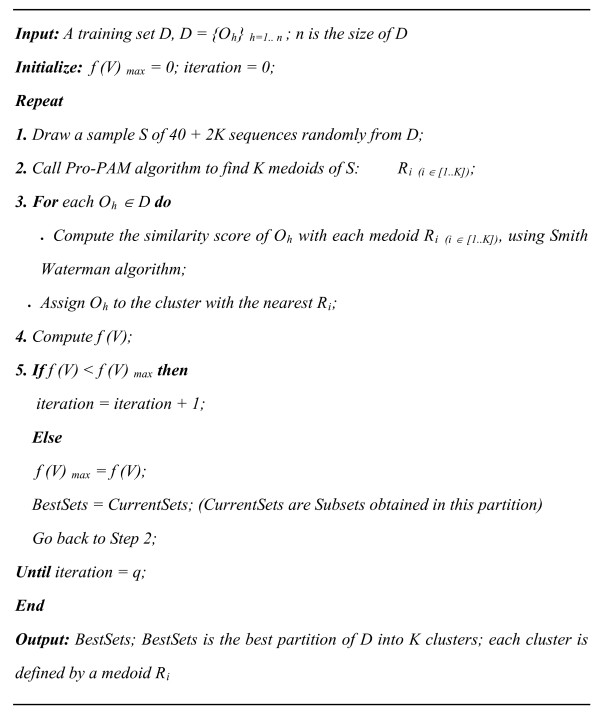
**Pseudo code for Pro-CLARA algorithm**.

Pro-CLARA uses the optimal set of medoids *R*_*i*__(*i *∈ [1..*K*]) _obtained by Pro-PAM and the Smith Waterman algorithm to compare each protein *O*_*h *_of the data set *D *with all medoids *R*_*i*(*i *∈ [1..*K*])_, and to assign the sequence *O*_*h *_to the nearest cluster. In order to alleviate sampling bias, Pro-CLARA repeats the sampling and the clustering process a pre-defined number of times, *q*, and subsequently selects as the final clustering result the set of medoids with the maximal *f (V)*. Input parameters of Pro-CLARA algorithm are the number of clusters, *K*, and of iterations, *q*, and as outputs the algorithm returns the best partition of the training base *D *and the *K *medoids of the obtained clusters. Pro-CLARA algorithm is detailed in Figure 4.

#### Pro-CLARANS algorithm

Pro-CLARANS algorithm starts from an arbitrary node *C *in the graph, *C = [R*_1_, *R*_2_,..., *R*_*k*_*]*, which represents an initial set of medoids. Pro-CLARANS randomly selects one of *C *neighbors, C*, which differs by only one sequence. If the total score of the selected neighbour, *TS*_*ih *_(Equation (5)), is higher than that of the current node *TS'*_*ih*_, Pro-CLARANS proceeds to this neighbor and continues the neighbor selection and comparison process. Otherwise, Pro-CLARANS randomly checks another neighbor until a better neighbor is found or the pre-determined maximal number of neighbours to check, *Maxneighbor*, has been reached. In this study *Maxneighbor *is defined as proposed by [30]

(7)

Where the maximal number of neighbours must be at least a threshold value 250 or obtained using the number of clusters *K *and the number of sequences, *n*, in the data set as: 1.25%*K*(n-K).

#### Pro-CLARANS algorithm aims to maximize the total score, *TS*_*ih*_

**Figure 5:**
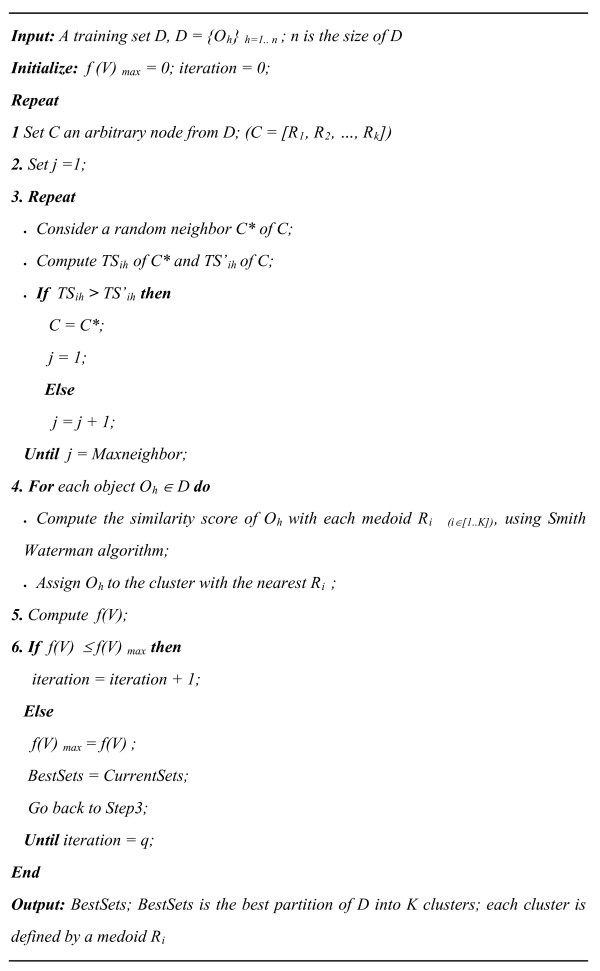
**Pseudo code for Pro-CLARANS algorithm**.

Pro-CLARANS algorithm use then the Smith Waterman algorithm to compute the similarity score of each sequence *O*_*h *_in *D *with each medoid *R*_*i*(*i *∈ [1..*K*]) _and to assign it to the nearest cluster. The algorithm repeats the clustering process a pre-defined number of times, *q*, and selects as the final clustering result the set of medoids with the maximal *f (V)*. Input parameters of Pro-CLARANS algorithm are the number of clusters, *K*, and of iterations, *q*, and as outputs the algorithm returns the best partition of the training base *D *and the *K *medoids of the obtained clusters. Pro-CLARANS algorithm is detailed in Figure 5.

The proposed algorithms presented here have been implemented in Java package. All of these algorithms used the EMBOSS  implementation of the Smith and Waterman local alignment algorithm for computing alignment score.

BLOSUM62 (Blocks Substitution Matrix) [35] was chosen to compute amino acids substitution scores [36]. We chose the default penalties proposed by Smith and Waterman EMBOSS implementation as gap opening (*P*_*o*_) and gap extension penalties (*P*_*e*_) (*P*_*o *_= 10 and *P*_*e *_= 2).

#### Performance measure

To evaluate the Pro-Kmeans, Pro-LEADER, Pro-CLARA and Pro-CLARANS clustering algorithms, a large data set, Training data set, is used. We obtained from the training phase *K *clusters and each cluster is defined by a medoid (centroid or leader). The training phase results are used to cluster a different data set named, Test data set. Smith Waterman algorithm is used to compare each protein sequence on the test data set with all medoids *R*_*i*(*i *∈ [1..*K*]) _obtained from the training phase, and to assign each sequence to the nearest cluster. The predicted family group of each sequence is which of the nearest medoid.

The results obtained from the test phase are used to calculate the *Sensitivity *and the *Specificity *of each algorithm and to compare them with results of the published clustering tools, ProClust, TribeMCL and JACOP, tested on the same set: "Test data set".

*Sensitivity *specifies the probability of correctly predicting a classifier and it is defined as

(8)

and *Specificity *the probability that the provided prediction is correct and it is defined as

(9)

where *TP *(True Positives) is the number of correctly identified true homologues pairs, *FN *(False Negatives) is the number of not identified true homologues pairs and *FP *(False Positives) is the number of non-homologue pairs predicted to be homologue. A pair of sequences is considered truly homologous, if both are in the same family group.

#### Protein sequence data sets

To evaluate the performance of the proposed clustering algorithms Pro-Kmeans, Pro-LEADER, Pro-CLARA and Pro-CLARANS, and to compare their results with the available graph-based clustering tools ProClust and TribeMCL and the only available partitioning clustering tool JACOP, protein sequence families with known subfamilies/groups are considered. Protein sequences of HLA protein family have been collected from . From this set, we have randomly selected 893 sequences named DS1 and grouped into 12 classes. Protein sequences of Hydrolases protein family have been collected from . Hydrolases protein family sequences are categorized into 8 classes according to their function and 3737 sequences, named DS2, have been considered from this family. From Globins protein family [37], sequences have been collected randomly from 8 different classes and 292 sequences, named DS3, have been selected from the data set *IPR000971 *in . Thus, totally 28 different classes containing sequences are considered as they have been classified by scientists/experts.

The data set considered has a total of 4922 sequences, named DS4, out of which 3500 sequences (practically 70% of the dataset DS4) are randomly for training, and 1422 for testing (practically 30% of the dataset DS4). The same method to obtain the training and the test sets are used on DS1, DS2 and DS3: randomly 70% of the set is selected for the training set and 30% for the test set [see Additional file 1].

## Results and Discussion

Experiments are conducted on Intel Pentium4 processor based machine, having a clock frequency of 2.4 GHZ and 512 MB of RAM. Experimental results are obtained using default values as follows. In Pro-Kmeans, Pro-CLARA and Pro-CLARANS algorithms, the number of iterations *q *is fixed to 5 [30] and the number of clusters *K *is fixed to 28. After a number of simulations, we find that the best clustering results are obtained when the parameter *K *= 28 [38]. In Pro-LEADER algorithm, the *Threshold *value is fixed to 350 after a number of simulations [28].

Experimental results of Pro-Kmeans, Pro-LEADER, Pro-CLARA, Pro-CLARANS, ProClust, TribeMCL and JACOP algorithms on DS1, DS2, DS3 and DS4 are summarized in Table 2.

**Table 2:** Performance of the three other tools (ProClust, TribeMCL and JACOP) and our four proposed methods on DS1, DS2, DS3 and DS4 data sets with respect to two clustering quality measurements: Sensitivity (Sens.) and Specificity (Spec.)

Algorithms	DS1		DS2		DS3		DS4	
	
	Sens.	Spec.	Sens.	Spec.	Sens.	Spec.	Sens.	Spec.
ProClust	50.64	56.77	48.71	61.86	46.09	55.14	46.39	51.07
TribeMCL	46.09	52.89	41.42	52.14	41.04	47.48	51.22	56.46
JACOP	99.92	66.27	99.96	70.06	99.96	73.96	99.92	94.42
Pro-Kmeans	92.38	99.90	55.32	98.01	63.30	96.92	56.06	99.56
Pro-LEADER	90.21	91.40	53.15	91.24	52.96	74.06	23.34	95.70
Pro-CLARA	93.60	99.92	73.28	99.26	81.53	98.60	77.84	99.66
Pro-CLARANS	93.10	99.90	78. 62	98.70	76.24	97.34	62.06	99.09

DS4 is a very large data set which contains all sequences of DS1 (HLA protein family), DS2 (Hydrolases protein family) and DS3 (Globins protein family).

In our experiments, the use of partitioning clustering methods Pro-Kmeans, Pro-LEADER, Pro-CLARA, Pro-CLARANS and JACOP have improved sensitivity and specificity of hierarchical methods, ProClust and TribeMCL.

We have demonstrated the performance of the proposed Pro-Kmeans, Pro-LEADER, Pro-CLARA, Pro-CLARANS algorithms for the clustering of protein sequences using similarity.

Experiments show also that on the considered data sets DS1, DS2, DS3 and DS4, the higher probability of correctly predicting a classifier (*Sensitivity*) is obtained using JACOP method. Pro-CLARA method gives the higher probability that the provided prediction is correct (*Specificity*) although that, Pro-Kmeans, Pro-LEADER, and, Pro-CLARANS obtain also good results. The use of Pro-LEADER method on very large and heterogeneous set, DS4, is not very valuable. In fact the number of not identified true homologues pairs (False Negatives) is very important for that, the obtained *Sensitivity *is limited to 23.34.

Pro-Kmeans, Pro-LEADER, Pro-CLARA and Pro-CLARANS result confirm that those proposed partitioning methods are valuable, reliable tools for the automatic functional clustering of protein sequences. The use of these methods instead of alignment methods or the classic known clustering methods by biologists can improve the clustering sensitivity and specificity and reduce significantly the computational time. The proposed methods can be used by new biologists especially to cluster a large data set of proteins into meaningful clusters in order to detect their functions.

## Conclusion

Similar protein sequences probably have similar biochemical function and three dimensional structures. If two sequences from different organisms are similar, they may have a common ancestor sequence and hence they are said to be homologous. Protein sequence clustering, using Pro-Kmeans, Pro-LEADER, Pro-CLARA and Pro-CLARANS methods helps in classifying a new sequence, retrieve a set of similar sequences for a given query sequence and predict the protein structure of an unknown sequence. We noticed that the classification of large protein sequence data sets using clustering techniques instead of only alignment methods reduce extremely the execution time and improve the efficiency of this important task in molecular biology.

## Competing interests

The authors declare that they have no competing interests.

## Authors' contributions

SF incepted and directed the research and wrote the manuscript. NE and ML participated in the coordination and the direction of the whole study. All authors read and approved the final manuscript.

## Supplementary Material

Additional File 1 **Training and test data sets**. The file contains text files which correspond to the used data set in this study in fasta format. The file contains two directories: the training base which has 3500 sequences and the test base which has 1422 sequences. The considered data set, named DS4, has a total of 4922 sequences out of which 3500 sequences (practically 70% of the dataset DS4) are randomly selected for training, and 1422 for testing (practically 30% of the dataset DS4). This dataset contains proteins selected from HLA (DS1), Hydrolases (DS2) and Globins (DS3) protein families. Click here for file
